# Supporting Young Children’s Physical Development through Tailored Motor Competency Interventions within a School Setting

**DOI:** 10.3390/children11091122

**Published:** 2024-09-13

**Authors:** Ellie Huggett, Kristy Howells

**Affiliations:** Department of Sport, Exercise and Rehabilitation Sciences, School of Psychology and Life Sciences, Canterbury Christ Church University, Canterbury CT1 1QU, UK; kristy.howells@canterbury.ac.uk

**Keywords:** motor competency, physical development, motor skills, young children, fine motor skills, gross motor skills, core strength and coordination

## Abstract

*Background/Objectives:* This study investigated how tailored motor competency (MC) interventions within a bespoke Scheme of Work (SOW) can support young children’s physical development (PD) by enhancing fine motor skills (FMS) and gross motor skills (GMS). The objective was to enable children to meet curriculum developmental physical milestones. *Methods:* The longitudinal case study design was conducted over 19 weeks across three academic terms and focused on 25 children (12 boys and 13 girls aged between 5 and 6 years old). Interventions within the SOW were evaluated at six points to assess effectiveness and to make adjustments. Data collection included observations on FMS and GMS development. *Results:* Statistically significant improvements were observed in FMS and GMS after implementing the MC interventions. Notable improvements included hopping, running, climbing, kicking, catching, and using scissors (*p* < 0.05). *Conclusions:* The study underscores the importance of holistic approaches to PD, highlighting the need for early intervention and the crucial role of educators. The findings advocate for strategically planned MC interventions and practitioner observations to achieve longitudinal improvements in PD. The study recommends nationwide implementation to enhance PD outcomes, preparing children for lifelong and life-wide MC.

## 1. Introduction

The purpose of this study is to investigate how motor competency (MC) interventions can help to support young children’s physical development (PD) through the creation of bespoke Schemes of Work (SOWs). These aim to develop both fine motor skills (FMS) and gross motor skills (GMS) alongside core strength and coordination to provide the children with the opportunity to meet their developmental milestones.

Early childhood physical activity (PA) engagement alongside physical and motor development are the cornerstones of long-term psychological and physical well-being [1]. According to research originating in London, early PA is influenced by the child’s age and physical ability, and by engaging in this PA, children are able to achieve significant developmental milestones earlier than other individuals who do not engage in the same quantity of PA [1]. A study undertaken in Canada examines the effects that children’s poor hand–eye coordination can have on a variety of activities, explaining that this can cause issues with learning to read and write as well as with playing sports since it makes it difficult to hit or catch a ball [2]. Additionally, difficulties with academic challenges might result from inadequate hand–eye coordination, as it may affect an individual’s ability to pay attention [2].

It is highlighted [3] that within England, children’s developmental stages trailed behind those expected for their age. Therefore, many young children were failing to meet age-related PD expectations demarcated in early learning goals (ELGs), which map out the developmental milestones within the curriculum of the Early Years Foundation Stage (EYFS) for practitioners [4]. The current curriculum suggests that there is motor potential for young children. However, previous research [3] highlights that young children are failing to meet age-related expectations regarding their overall motor development of FMS and GMS. Further highlighting the necessity for tailored support plans, which strive to develop both FMS and GMS to upskill the children to meet developmental targets, a systematic research review demonstrated that more engagement in PA contributes to improved MC and cognitive development in the early years [5,6]. To do this, educators required both guidance and assistance on how to best involve children in a wide range of captivating activities that further develop PD in the impacted areas.

As mentioned above, FMS and GMS are crucial for future PA levels since participation in PA during the younger years can promote the development of FMS and GMS, which, in turn, enhance engagement in life-long PA [7,8]. Evidence also suggests that FMS and GMS play an important role in school readiness (SR) [9]. However, only 65.2% of children in England achieve a good level of PD by the end of their first year of school [10]. Jones et al. [9] gathered data from 326 four- and five-year-old children in the northeast of England for a cross-sectional investigation to examine the association between being physically active, having ‘good’ FMS and GMS, and being school-ready. According to a regression analysis of their findings, motor-skill characteristics and sedentary behaviour were strongly predictive of SR, unlike PA [9]. They continue to explain that sedentary behaviour and motor skills both strongly influence SR and that, as a result, encouraging motor skills and developmentally suitable sedentary behaviour activities may improve the number of young children who are deemed school-ready.

Previous research [3] states that children require a tailored plan that attempts to assist in strengthening both their FMS and GMS to drive them to reach developmental milestones before moving into the more formal stages of education within England, referred to as Key Stage 1, highlighting why this study is important. It has been previously argued that the children are not developmentally ready to transition into Key Stage 1 [3]. Therefore, this study acts upon these previous recommendations [3] and develops bespoke SOWs based on recently released government guidance [11], specifically aiming to develop the skills in which previous research found the children to be developmentally behind. This kind of intervention was designed to promote positive changes within motor development, and it was expected that this holistic approach would improve both FMS and GMS.

## 2. Materials and Methods

### 2.1. The School and Sample

A total of 25 children (6 boys aged 5 and 6 boys aged 6, average age 5.5 years, SD ± 0.3 years; 8 girls aged 5 and 5 girls aged 6, average age 5.38 years, SD ± 0.49 years) from a rural village faith (Church of England) primary/elementary school in the southeast of England participated in this study. The children come from a variety of backgrounds due to the location of the school, with many coming from social housing and gated communities. All children were included in this study; it was a whole class approach.

Purposive sampling techniques can help with ensuring studies are rigorous and trustworthy [12]. It is acknowledged that both purposeful and convenient sampling techniques were used within the study. They were purposeful, as the sample within this study were the same children who undertook previous research with the lead researcher [3] and who were found to have not yet met their PD goals and were in need of support, as recommended earlier. The previous study, Huggett and Howells, [3], suggested the idea of tailored interventions; this study is the research of those tailored interventions. The research is also convenient, as the sample consisted of children from the school where the lead researcher worked as the Head of Physical Education, Health and Wellbeing and had easy access to and contact with them daily. It is possible that it could be perceived that the lead researcher is biased towards seeing improvements in MC and PD; however, a research team was used in the data collection to reduce any bias.

The bespoke SOW was a whole-class intervention and was not tailored for sex. This was following previous researchers for this age group, who found that there is an absence of noticeable disparities when comparing young boys and girls in terms of FMS and GMS [13,14].

### 2.2. The Timeline of the Study

**Figure 1:**
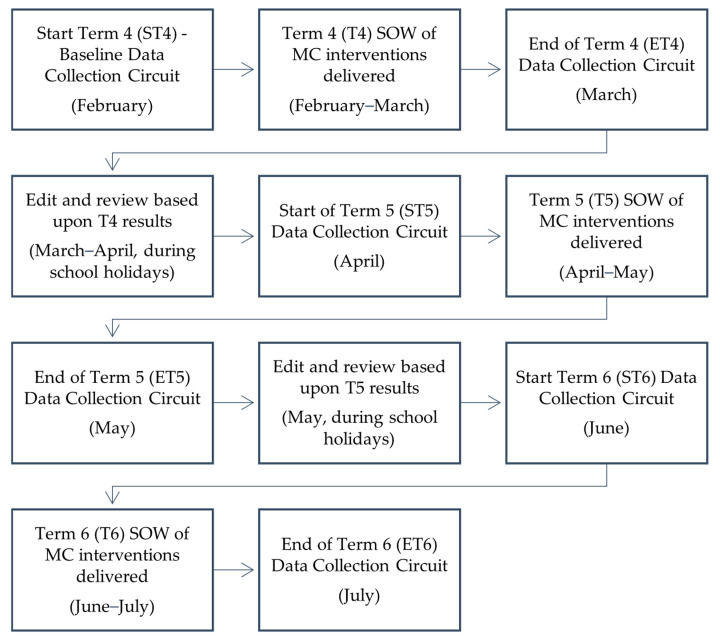
The timeline of the study.

The longitudinal case study design allowed for a 19-week analysis (February–June) across three academic terms. The timeline below (see Figure 1) demonstrates how and when data were collected. Baseline data were gathered at the start of term 4 (ST4), the SOW was then delivered during term 4 (T4), and further data were later collected at the end of term 4 (ET4). This made it possible to pinpoint progressions or regressions that transpired throughout the duration of T4’s SOW or the school holidays. The subsequent SOW (for terms 5 and 6) could then be modified with the intention of developing the PD components that the children were either deficient in or showing only minor development in, making it an iterative process that changed depending on the needs of the children. The same procedure was then repeated for terms 5 (T5) and 6 (T6).

### 2.3. Observations as Research Methods

**Figure 2:**
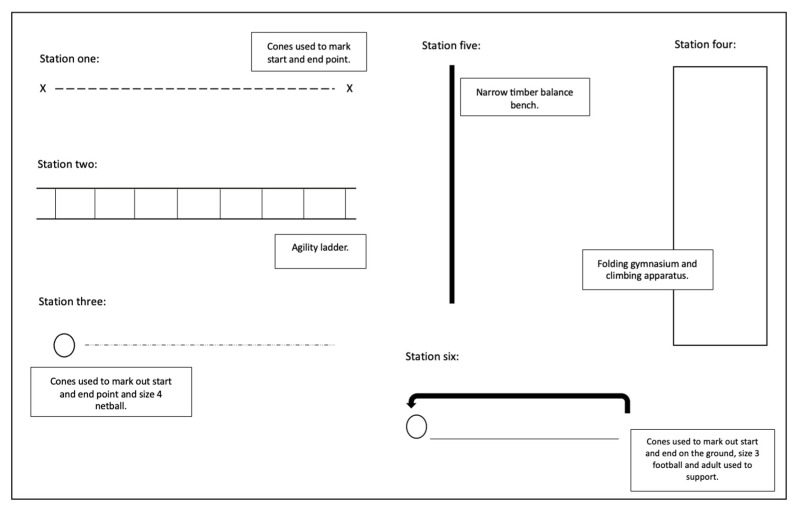
The assessment circuit.

This assessment circuit (see Figure 2) was used to standardise the observations that focused on GMS, providing minimal opportunities for FMS to be observed. FMS data, in particular the children’s use of scissors, were collected during explicit sessions tailored explicitly to observe the children’s use of scissors.

The observations were a multi-team effort to enable all children to be observed, and other practitioners supported the process. To ensure that the observations from the team were reliable, prior to data collection, ‘mock circuits’ were used, which acted as a training course for other practitioners participating in the observational assessments. Training such as this is vital for practitioners who are involved in research and is critical since they can have a major impact on the success of studies [15]. This form of ‘careful study planning’ [16] was adopted as previously recommended as a rigorous approach. This strategy enabled opportunities to practice using the data collecting sheets to ensure precisely what each success criteria (SC) of the skills looked like in practice. This consequently minimised any disparities in the observations of each criterion to ensure that consistency across the data collection process was upheld regardless of which adult gathered the data [17].

### 2.4. Assessment Criteria for Each of the Skills

For the development of the assessment criteria, this study used and adapted the criteria from subtests of the second edition of the Test of Gross Motor Development (TGMD-2) [18] for the skills of hopping, running, and kicking. Each of the skills (hopping, running, kicking, climbing, and the use of scissors) was split into 4–5 SC needed to execute the skill effectively. The children were tested against these criteria at the beginning and end of each term. The SC can be found below.

The TGMD-2 [15] was used and adapted (see Table 1), as it is defined as a standardised norm and criterion-referenced test [19] comprising two gross motor development subtests, object and locomotor control, each of which has six tasks that measure a distinct element of gross motor development [18]. A review [20] analysed the test substance, validity, and normative samples of seven movement skill assessment tools, such as the TGMD-2, and found that TGMD-2 does not examine FMS or stability movement skill development. Although the TGMD-2 is ideal for measuring GMS and used within this study, alternate assessment criteria created from the age-related expectations found within the Development Matters Documents [21] and the ELGs within the EYFS curriculum document (Department for Education, [22]) were used to examine FMS development in addition to GMS development. The initial aim of the ELGs, found within the EYFS document [22], is to accommodate a straightforward and effective transition for children into KS1 by presenting knowledge on each child’s learning needs and development to the new teacher. These EYFS guidelines were created to provide a framework for delivering consistent and high-quality environments for children, recognising the importance of this age in a child’s development [21,22].

A good assessment technique should not only target the domain of concern but be trustworthy, reliable, simple to administer, and easy to accommodate change [23]. This case study employed an adapted version (see Table 1) of the assessment criteria previously used to assess balance in America [24]. To measure balance and core motor control, they employed simplified adaptations of the standard one-leg balance tests conducted on both steady and unsteady surfaces. This research examined and documented the capacity of the participants to maintain balance [24]. They were scaled from one (representing “normal” balance) to five (being an abnormal balance requiring a step down).

The assessment circuit was designed for the children to walk across a balance beam steadily and carefully, stopping in the middle. As the children were not asked to do a simple one-leg balance, like the previous researchers [24], it was necessary to adapt and modify their 5-point scale criteria to just 3 SC.

The assessment criteria for the use of scissors have been centred on age-associated PD needs outlined in the Development Matters document [21] and the EYFS [22], again using point-scale criteria, this time 4 SC (see Table 1).

### 2.5. Motor Competency Interventions within the Scheme of Work (SOW)

Research on PA promotion and preservation during childhood persistently shows that ‘multi-component, multi-modal, and multi-outcome’ interventions are the most effective [25,26]. Therefore, the interventions within the bespoke MC SOWs were designed to include a wide range of engaging activities and meet the PD needs of the children to have a greater effect, as they target a variety of PA modalities.

The main focus of motor development interventions, such as those employed throughout terms 4, 5, and 6, which are found in Table 2 below, is on strategies that offer sufficient instruction with the sole objective of acquiring motor skills [27]. A recent systematic review [28] put a particular emphasis on the efficiency of school-based interventions for young children between the ages of 3 and 12. The study concluded that while school-based treatments generally had favourable results, the magnitude of the effect varied depending on the type of intervention [28]. By creating MC interventions within the SOWs that focused on learning, practising, and repeating motor skills [29], the recent suggestions made by the Department for Education [11] in their ‘help for early years providers’ document were also considered when developing the bespoke SOWs.

Alongside the explicit MC interventions found within the SOW, warm-up activities (see Table 3) that specifically targeted the targeted skill development were employed. These warm-up activities are designed to help children develop their spatial and positional awareness, core strength, and coordination, which were identified as key areas where these children did not perform at the predicted levels in earlier studies [3].

### 2.6. Statistical Analysis

SPSS 24.0 statistical analysis (IBM Corp, Armok, NY, USA) was used. To determine if there were changes throughout the SOW and tailored MC interventions, the Wald chi-square statistic was used. The significance threshold was set at <0.05, and pairwise comparisons were employed to determine where these differences were throughout the scheme.

## 3. Results

### 3.1. Hopping

**Figure 3:**
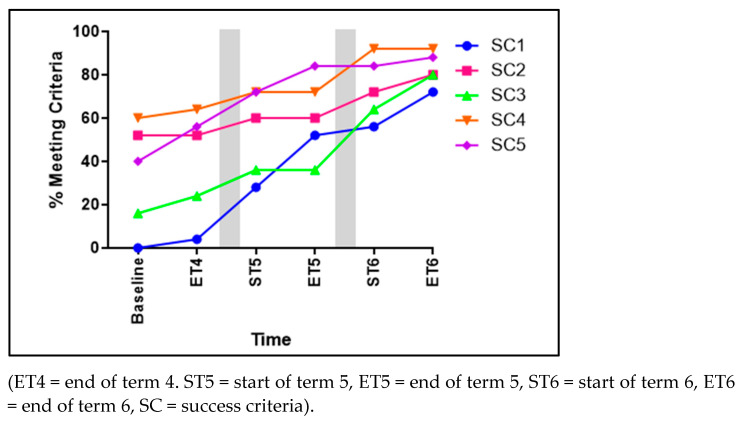
The percentage of children meeting each of the five success criteria for hopping.

Significant improvements in hopping were evident for the children over time for four of the five SC (see Figure 3). These included the following: non-support leg swinging forward in pendular fashion to produce force (SC1) significantly improved to 75% of the children achieving this from baseline to ET6 (χ^2^ 17.091, *p* < 0.001). The foot of the non-support leg remaining behind the body (SC2) significantly improved from 52% to 80% of children achieving this (χ^2^ 8.620, *p* = 0.035). Arms flexed and swing forward (SC3) significantly improved to 64% of the children achieving this across the three terms (χ^2^ 22.730, *p* < 0.001). Taking off and landing three consecutive times on the non-preferred foot (SC5) also significantly improved to 48% (χ^2^ 18.756, *p* = 0.002) of the children achieving this skill. Contrarily, SC4, taking off and landing three consecutive times on the preferred foot, showed no significant advancements (χ^2^ 7.786, *p* = 0.051).

### 3.2. Running

**Figure 4:**
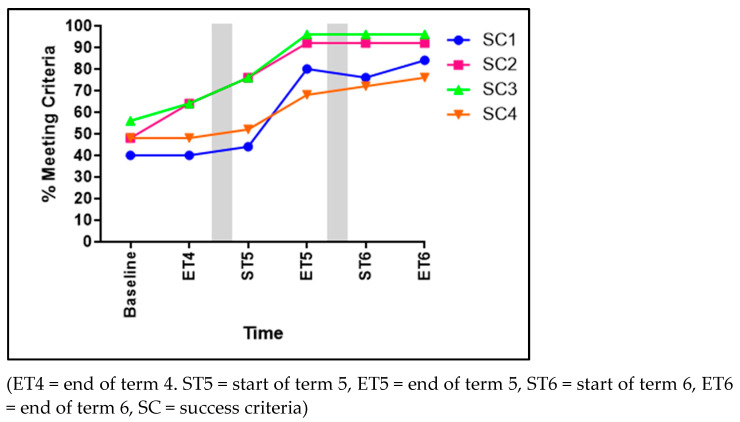
The percentage of children meeting each of the four success criteria for running.

Significant improvements were identified within three of the four SC for running (see Figure 4). These improvements can be seen in the following criteria: arms moving in opposition to legs (SC1) increasing by 44% from baseline to ET6 (χ^2^ 14.561, *p* = 0.006), a brief period where both feet are off of the ground (SC2) significantly improved from 48% to 92% (χ^2^ 13.216, *p* = 0.04), and narrow foot placement landing on heel or toe (not flat-footed) (SC3) significantly improved by 40% over the course of the three terms (χ^2^ 9.957, *p* = 0.019). However, SC4 (non-support leg bent at approximately 90 degrees) revealed no significant developments (χ^2^ 8.677, *p* = 0.070).

### 3.3. Kicking

**Figure 5:**
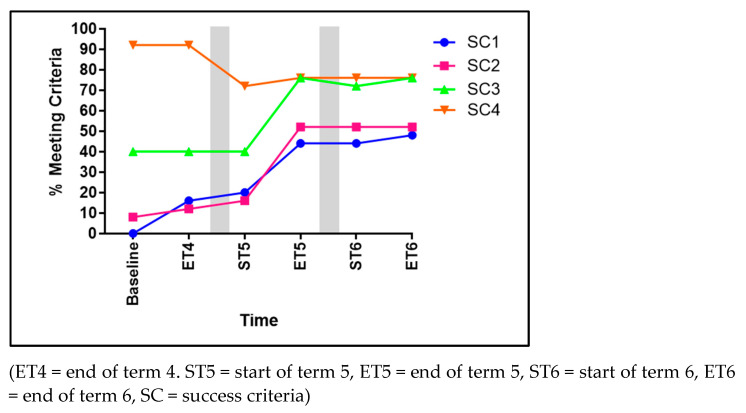
The percentage of children meeting each of the four success criteria for kicking.

A total of three out of four SC were identified to have had significant advancements within the skill of kicking (see Figure 5). These advancements can be seen in the following criteria: rapid continuous approach to the ball (SC1) significantly improving by 48% from baseline to ET6 (χ^2^ 9.887, *p* = 0.020), an elongated stride or leap immediately prior to ball contact (SC2) significantly improving from 8% to 52% (χ^2^ 14.381, *p* = 0.006), and the non-kicking foot placed even with or slightly behind the ball (SC3) significantly improving by 36% (χ^2^ 12.353, *p* = 0.002). The final criterion, kicking the ball with the instep of the preferred foot (shoelaces) or toe, was found to have no significant effect (SC4) (χ^2^ 3.297, *p* = 0.192).

### 3.4. Climbing

**Figure 6:**
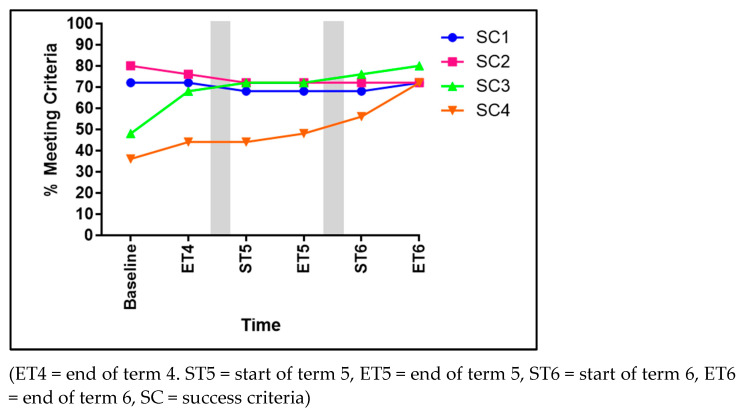
The percentage of children meeting each of the four success criteria for climbing.

Significant advancements were evident within two of the four SC for climbing (see Figure 6). These significant advancements can be seen in mainly using over grip and closed grip (SC3), which significantly improved by 32% from baseline to ET6 (χ^2^ 11.544, *p* = 0.042), and the use of diagonal reciprocal movement activation pattern (SC4) significantly improving from 36% to 72% (χ^2^ 12.611, *p* = 0.013). Contrarily, no significant effect of time was found within SC1, climbing rhythmically (χ^2^ 1.039, *p* = 0.308), or in SC2, observing only the direction of climbing (χ^2^ 2.147, *p* = 0.342).

### 3.5. Use of Scissors

**Figure 7:**
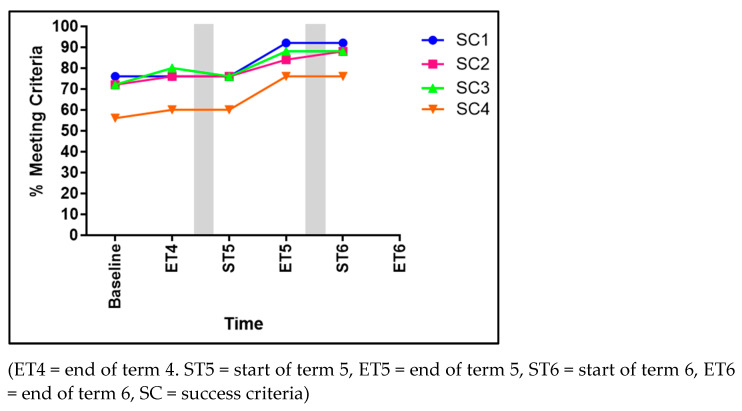
The percentage of children meeting each of the four success criteria for the use of scissors.

Within the four criteria for the use of scissors, only one criterion had significant development: to cut in a straight line (SC3) significantly improved by 16% from baseline to ET6 (χ^2^ 14.543, *p* = 0.006) (see Figure 7). Meanwhile, no significant effects of time were evident within the other three criteria: holding scissors in the dominant hand with the correct fingers (SC1) (χ^2^ 4.182, *p* = 0.124), opening and closing the scissors when cutting as opposed to tearing the paper (SC2) (χ^2^ 6.839, *p* = 0.145), and rotating the paper whilst cutting (SC4) (χ^2^ 5.802, *p* = 0.055). It should be acknowledged that the last data collection point (at the ET6) was missed for this skill due to the complex nature of early educational settings. The data for the four SC within this skill were gathered until the ST6.

## 4. Discussion

### 4.1. Gross Motor Skills Development

GMS development is crucial for the development of healthy bodies, as well as social and emotional well-being [22]. The SOW and the MC interventions within it (see Table 2 above) sought to develop these GMS through not only indoor but also outdoor activities, as they are commonly acknowledged to be a key aspect of development for young children that is often overlooked [30,31]. The following discusses how the SOW helped to support each of the skills throughout the academic year.

#### 4.1.1. Hopping

Within the skill of hopping, accelerated progress is evident within SC1: non-support leg swings forward in pendular fashion to produce force. Progress was identified over each of the three terms, but the most progress occurred in T5 and T6. To execute this SC effectively, the children needed adequate leg strength and power to generate force during the hop. The muscles of both legs, particularly the quadriceps, hamstrings, and calf muscles, needed to contract forcefully to propel their bodies upward and forward. Within the SOW, there are many interventions designed to support the development of leg strength required for the successful execution of this SC, such as:

*Gardening*—Gardening encourages and promotes children’s PD muscular strength, FMS, and coordination [4]. Within the SOW, this involved squatting, bending, and lifting when doing tasks such as planting, weeding, and moving bags of soil. These repeated motions can gradually develop the children’s leg muscles, especially the quadriceps, hamstrings, and glutes. Additionally, the joints are reinforced and are therefore made increasingly more flexible. Children, in particular, like the act of digging holes, planting plants in them, and then pushing the soil down to ensure they are in place. Movements like this help to improve core strength and coordination.

*Climbing*—within the SOW, this involved pushing and pulling with the leg muscles to propel the body upward and support body weight. It engages muscles like the quadriceps, hamstrings, calves, and glutes while also challenging balance and stability.

*Building an obstacle course*—van Hyfte et al. [32] explain that repeated engagement with obstacle courses can lead to improved MC. Therefore, within the SOW, this involved building and manoeuvring through the obstacle course, which is proposed to aid in developing leg strength. The children were actively encouraged to incorporate tasks such as jumping over equipment, climbing, crawling under obstacles, and running between stations—all of which develop lower body strength.

Furthermore, the regular modelling and repetition of the skills [33] throughout the assessment circuits contributes to the progression identified within this SC. As explained by Kajanus [34], the repetition of the physical skill leads to the mastery of the skill.

#### 4.1.2. Running

Running involves a repetitive sequence of movements, including lifting the legs, swinging the arms, and propelling the body forward, all of which require GMS to coordinate the large muscle groups in the legs, hips, and core to generate propulsion and maintain momentum. Running also requires adequate muscle strength and power to lift the body off the ground during each stride. SC2 within the skill of running involved the children having both feet off the ground for a brief period of time. This requires well-developed GMS, as the muscles in the legs, particularly the quadriceps, hamstrings, and calf muscles, generate the force for propulsion. It should be noted that although GMS are vital for the execution of this SC, so is dynamic balance. During the baseline assessment, 48% of children were meeting SC2, which rose to 68% by the end of T4. This figure further rose upon the return from the school break at the beginning of T5, from 64% to 76% at the end of T5, rising to 92% by the end of T6. In each of the terms, there were many interventions that specifically targeted the development of locomotion-focused GMS and consequently contributed to the increase in the percentage of children meeting SC2 over the three terms, some of which are detailed below:

*Building obstacle courses*—Play activities with a purpose, such as obstacle courses, motivate children to engage in PA while developing their muscles [35]. Within the SOW, this involved the children setting up obstacle courses, and they were actively encouraged to include activities such as crawling, jumping, and climbing. Crawling is a fundamental movement pattern that engages muscles in the legs, hips, and core. For example, the children crawled through tunnels and under obstacles within the courses, which contributed to the development of both coordination and strength in the lower body muscles. The children also jumped over hurdles and leapt across gaps between equipment, which challenged the lower body muscles to generate power and force. These jumping and crawling exercises helped to develop strength and coordination in the legs, which is needed to execute the SC2 of running effectively.

*Adventurous climbing and swinging*—Ridgers et al. [36] suggested that when young children spend increased time outdoors, this often correlates with increased PA and, therefore, increased motor development. Therefore, within the SOW, this involved the children participating in both indoor and outdoor adventurous climbing and swinging. Climbing and swinging require significant engagement of the muscles in the legs, including the quadriceps, hamstrings, glutes, and calves. These muscles work together to generate force and propel the body upwards or forwards during climbing and swinging movements, therefore placing resistance on the lower body muscles and promoting strength development. Additionally, the repeated contraction of leg muscles during climbing or the force generated to push off and swing forward when swinging helped the children to strengthen muscles throughout their lower body.

*Gardening*—within the SOW, this involved lifting and carrying soil, the navigation of uneven terrain, pushing and pulling weeds, and using a shovel or spade to dig holes, turn soil, or plant seeds, all of which require lower body strength and coordination. The pushing and lifting motions involved in digging engage muscles in the legs, contributing to running development.

#### 4.1.3. Climbing

Climbing involves the coordinated movement of the children’s entire bodies, including the arms, legs, torso, and core muscles. For the children to perform these intricate movements, which include reaching, pulling, pushing, and stepping to scale the climbing wall, developed GMS were necessary. The overall percentage of children executing the skill of climbing rose from 36% during the baseline assessment to 72% at the ET6. Climbing relies heavily on upper body GMS due to the significant involvement of the arms, shoulders, and upper back muscles in pulling and supporting the body weight during ascent. The repetition of interventions that target the explicit development of GMS contributed to the huge improvements seen across the three terms. As the DfE [11] explains, weight-bearing activities such as lifting and repositioning large items or hanging and swinging from climbing equipment contribute to the development of upper-body strength [11].

Furthermore, the children have access to climbing facilities every day outside on the playground, with the potential to promote PD due to the rich affordance landscape [37]. However, it is important to consider that just because this activity could specifically target GMS development, this does not necessarily mean that the children will immediately recognise this and take advantage of it [38]. However, as Head of Physical Education, Health and Well-being, it was the responsibility of the research lead to ensure that children had the opportunities to engage in adjacent environments at school and in sporting situations [37].

### 4.2. Fine Motor Skills Development

The sooner children have the opportunity to develop their FMS through proper instruction and practice opportunities [39], such as those found within the SOW of this study, the better their self-esteem, academic success [40], and long-term health outcomes [41,42]. This study sought to provide the children with a variety of rich chances for both big and small movements, as recommended by the DfE [11]. The foci for FMS within the SOW were climbing, the use of scissors, and the use of cutlery.

#### 4.2.1. Climbing

When climbing, children often need to make small adjustments to their grip on holds, especially when using an over grip, which is a grip required within SC3 for climbing. Dramatic improvements were identified within SC3—rising from 48% during baseline assessment to 68% by the end of T4. Well-developed FMS allow the children to make the subtle adjustments required to execute FMS with their hands to maintain a secure grip on the hold. This type of grip requires precise finger placement and tension control to maximise contact with the hold and distribute their body weight effectively. Over the course of T4 and T6, many interventions within the SOW targeted the development of FMS and tension control:

*Baking cookies and making playdough*—within the SOW, this involved the children baking their own cookies and making their own playdough. They kneaded and rolled the dough, which required varied levels of finger tension control. The children also squeezed and shaped the cookies and playdough, which helped them develop strength and dexterity in their fingers. Involving young children in food preparation and cooking on a regular basis is a highly motivating way of improving their FMS [11]. Cooking activities found within the baking of cookies and making of playdough, such as squeezing, mixing, pouring, and spreading, support the development of FMS and hand–eye coordination abilities [11]. Specifically, the motion of rolling and flattening a mixture, such as playdough, with the hands or with a rolling pin helps children practice utilising both hands in a coordinated manner. Furthermore, pouring ingredients into bowls is another effective method for developing hand–eye coordination.

*Adventurous outdoor climbing and swinging*—As Howells [43] explains, it is crucial to consider what is occurring within outdoor spaces to encourage physical activity and physical development; within the SOW, the children partook in climbing and swinging activities within an outdoor environment. This consisted of playground equipment, trees, and rope swings within the school grounds. Children must use mostly their hands and fingers to support their body weight as they climb and swing. Gripping onto holds, ropes, or bars provided a challenge for the muscles in the children’s fingers, hands, and forearms, gradually building grip strength and finger tension control over time. Children had to change their finger tension to suit the needs of each unique grasp on the variety of trees, climbing apparatus, and monkey bars, which included a variation of surfaces. This helped children acquire finely tuned control [43].

*Building obstacle courses*—within the SOW, this involved children being instructed to construct an obstacle course that included tasks that encourage the development of FMS, such as tying knots and securing ropes. These activities require precise finger movements and tension control to manipulate and fasten equipment in place. Within this intervention, the children also had the chance to climb over numerous objects, with an adult modelling the correct hand placement for this. This type of modelling is supported by Curtin [33], who explains that through modelling, educators can demonstrate the activity effectively and ensure proper form is employed by the children [33].

*Weaving*—within the SOW, this involved helping to enhance the children’s FMS by supporting them with grasping, establishing their pincer grip, and manipulating various materials [11]. The children began the session by folding their own ‘caterpillars’ and then continued to weave different coloured paper to form a pattern. It was important that within these sessions, educators checked for high levels of interest and observed each child’s dexterity, as this provided insight as to whether the materials chosen were appropriate and were providing a degree of challenge [11]. To guarantee that this level of difficulty was present for each child within this intervention, the children utilised varied-size strips of paper based on their ability.

As the children began to develop, learn, and improve a variety of FMS throughout these activities, their capacity to perform movement skills, such as climbing, increased rapidly [39]. Participation in activities that have a focus on FMS, such as the ones outlined above, is also crucial for children to develop coordination and regulation of their overall body movements [44].

#### 4.2.2. The Use of Scissors

Since using scissors requires hand–eye coordination and limb control, children who master it well will also benefit from improvements in other FMS [45]. SC1 within the skill of using scissors required the children to hold the scissors with their correct fingers in their dominant hand. This action of holding the scissors required FMS fundamentally because it involved the control and coordination of the children’s fingers and hand muscles to manipulate the scissors effectively. The children hold scissors with a precision grip, which entails grasping the handles of the scissors with their thumb, index, and middle fingers. For children to be able to precisely place their fingers and apply the proper amount of pressure to grasp the scissors firmly, they require well-developed FMS. SC1 saw its first increase in the percentage of children meeting it within T5, increasing from 75% at the beginning of T5 to 92% by the ET5.

Furthermore, SC2 within the skill of using scissors required the children to open and close their scissors when cutting paper, as opposed to ripping it. A steady progression over the course of T4 and T5 was identified within this SC, with 72% of children meeting the SC during the baseline assessment and rising to 88% at the ST6. Well-developed FMS enable the children to regulate the amount of pressure they apply to the scissors handles to ensure that the blades meet smoothly and cut through the paper without excessive force, avoiding tearing.

Both SC1 and SC2 require the children to be able to regulate the delicate control the children need to have over their finger muscles to achieve the desired cutting effect—ensuring that they use the correct amount of pressure to hold the scissors correctly and apply the correct amount of pressure when cutting. This is developed within interventions found within the SOW, such as:

*Using scissors*—within the SOW, this involved the children having explicit lessons in using scissors correctly. By ensuring children have explicit lessons modelling the correct amount of pressure, the children were able to work within small groups with adult support and guidance, developing their understanding of the amount of pressure to apply and when to apply it through discussion and modelling.

*Gardening*—By allowing children to manipulate and handle a variety of gardening instruments, gardening and associated tasks can enhance motor development. Therefore, within the SOW, the children trimmed plants with scissors and pruners [46]. The children were then guided by adults to apply the right amount of pressure to cut without damaging the plant. This activity helped the children to develop control over pressure regulation and FMS.

Within the SC2 for using scissors, the thumb is an essential component in the operation of the scissors, as it controls the opening and closing of the tool. FMS are required to synchronise thumb movement to manage the cutting process and smoothly operate the scissors. Within the SOW, the children participated in weaving, which involved them cutting strips of paper to weave. Engaging in crafting activities such as this that involve cutting paper allowed the children to practice using scissors, promoting skill development and precision.

#### 4.2.3. The Use of Cutlery

Within the SC4 for the use of cutlery, children are required to cut their food with a knife and fork. Cutting through different types of food requires varying levels of pressure. FMS allow the children to adjust the pressure exerted on the knife according to the food’s texture. During the baseline assessment, 37.5% of the children met SC4, and by the ET6, that figure rose to 75%. This is thought to be due to the extensive number of interventions within the SOW that aim to develop FMS, which helped to develop the skills of using cutlery.

## 5. Strengths and Limitations

This study is timely, as it examines the impact of the implementation of governmental guidance on early years PD [11], which suggests the need for tailored and focused interventions on FMS, GMS, and core strength and coordination. It is also timely as it continues to follow the same children who were previously identified as being behind in their PD and in need of interventions to support their overall development. A particular strength of this study is the novel approach to following the children’s progression over a longitudinal period of 19 weeks to analyse the impact of MC interventions from the bespoke SOWs. It does not just provide a snapshot but also considers the effectiveness of the activities within the SOW and how these activities helped improve the children’s motor skills. The aim of this analysis was to help future practitioners see not only the how but also why they need to implement specific interventions.

It is acknowledged, though, that this study is limited due to focusing on one case study school. However, focusing on the MC interventions of SOW over a longitudinal time allowed for depth of data for this educational setting, isolating the variables of socio-economic background, curriculum, and the influence of affordances within the school setting. Although this study is limited in terms of generalisability beyond the population represented, the conclusions and data provide an important insight. The findings could inform a larger-scale study examining more schools nationally to then include a range of economic and geographical settings.

## 6. Conclusions and Recommendations

This longitudinal case study has illustrated that school can be a key place to help support and develop PD effectively. This study has shown that a bespoke SOW containing multiple engaging MC interventions aiming to develop the children’s PD is effective longitudinally over three academic terms. To optimise development, although the SOW and MC interventions within it are critical to the development of PD levels, it is imperative that these be combined with effective PE lessons and ample opportunities to engage in PA and sports both inside and outside of school, as well as during recess time. Moreover, it is crucial that practitioners and teachers aim to develop each child holistically and consider their unique differences, and this can be undertaken through a whole-class approach, as this study has shown.

### Recommendations

The results of this study highlight the need for strategically planned MC interventions focusing on PD coupled with practitioner observations. As this study has demonstrated the possibility of significant longitudinal improvements across various aspects of the development of FMS and GMS, it is recommended that this should be implemented nationwide to improve PD outcomes and ensure that our young children are equipped with lifelong and life-wide skills to continue their unique physical activity journeys. It is proposed that MC interventions and PD can be applied globally within educational settings, as all children need FMS and GMS and are, therefore, not limited to just the English curriculum. For future research, we would recommend that international comparisons of motor development and FMS and GMS development are undertaken.

## Figures and Tables

**Table 1:** The success criteria required for hopping, running, kicking, climbing, and the use of scissors in order to execute the skill effectively.

Skill	SC1	SC2	SC3	SC4	SC5
Hopping	Does the child’s non-support leg swing forward in pendular fashion to produce force?	Does the child’s foot of non-support leg remain behind the body?	Are the child’s arms flexed and swing forward?	Does the child take off and land three consecutive times on preferred foot?	Does the child take off and land three consecutive times on non-preferred foot?
Running	Did the child’s arms move in opposition to their legs?	Was there a brief time when both of the child’s feet were off the ground?	Did the child have a narrow foot placement landing on their heel or toe (not flat-footed)?	Was the child’s non-support leg bent at approximately 90 degrees?	
Kicking	Did the child have a rapid continuous approach to the ball?	Did the child use an elongated stride or leap immediately prior to ball contact?	Did the child place their non-kicking foot even with or slightly behind the ball?	Did the child kick the ball with instep of preferred foot (shoelaces) or toe?	
Climbing	Does the child climb rhythmically?	Does the child observe only the direction of climbing?	Does the child use over grip and closed grip?	Does the child use diagonal reciprocal movement activation pattern?	
The use of scissors	Does the child hold scissors in their dominant hand with the correct fingers?	Does the child open and close the scissors when cutting as opposed to tearing the paper?	Can the child cut in a straight line?	Can the child rotate the paper whilst cutting?	

**Table 2:** Illustrative examples of the motor competency interventions found within the bespoke SOWs used within the study.

Motor Competency Interventions Found within the Scheme of Work
*Baking cookies.* The children will prepare the ingredients and roll and flatten dough with their hands, as well as mix, stir, squeeze, and spread the mixture. Finally, they will bake their cookies.
*Building an obstacle course using different terrains, such as boxes, hills, and beams.* Children will have the chance to build their own obstacle course. Once the obstacle course is constructed, children will revisit it. They will reflect upon their previous circuits, and the children will uplevel and improve their circuits and develop games around them. Each time, they will become increasingly skilful in negotiating the different spaces, pieces of equipment, and the length of the course. Differentiation will be provided by the change in pace and difficulty of each section of the course.
*Adventurous climbing and swinging—outdoor climbing and swing frames.* Children will partake in adventurous climbing on outdoor play equipment and trees within the school grounds.
*Adventurous climbing and swinging—indoor climbing and swing frames.* Children will utilise the indoor climbing apparatus, learning to climb a range of different mediums effectively using accurate foot and hand placement.
*Use of scissors.* Children will spend an entire lesson on the skill of cutting and how to use and move their hands.
*Moving house.* Children will wrap up items and place them in boxes—ready to move houses! The children will be required to physically pick up and move the boxes and ‘furniture’. Using clipboards, the children will make lists, checking that all possessions have arrived safely at their new house!
*Homemade playdough.* The children will prepare the ingredients and roll and flatten playdough with their hands, as well as mix, stir, squeeze, and spread the mixture. Afterwards, the children will mould their playdough into shapes.
*Weaving.* Children will be interlacing two sets of threads or yarns, using right angles to develop a ‘snake’. Afterwards, children will weave strips of paper together to create their own patterns.
*Gardening—weeding areas around the school and potting plants.* Children will participate in weeding the flower beds around the school grounds and then will plant plants.

**Table 3:** Illustrative examples of the warm-up activities used within the study.

Warm-Up Activities
*‘Traffic Lights’*—The children respond to orders like ‘red light’ and ‘green light’, which improves their spatial placement and control by forcing them to consider and analyse their location and stop or respond accordingly. Their ability to estimate distances and modify their pace to halt on schedule improves their timing, coordination, and spatial judgement.
*‘Stuck in the Mud’*—By navigating the space, children improve their understanding of and ability to navigate around their spatial surroundings. To avoid being caught, they must quickly change directions, which requires agility, positional awareness, and the ability to continuously observe the locations and movements of others. Later during terms 5 and 6, the children played ‘Stuck in the Mud’ with a twist. Once stuck, they held the crab position, and one of their team members had to roll a ball (striding forward, using the correct technique) underneath them to ‘unstick’ them. The incorporation of ‘the bridge’ into this game aimed to improve the children’s balance and core stability.
*‘Simon says’*—In this classic game, I gave commands such as “Simon says touch your toes” or “Simon says jump forward,” requiring the children to follow the instructions while being mindful of their position in space and the movements of others.
*‘Mirror movements’*—In pairs, the children face each other and take turns mirroring each other’s movements. This game promotes spatial awareness as the children are required to synchronise their movements whilst being aware of their own and their partner’s position.
*‘Animal crawls’*—This game encourages the children to mimic different animal movements, such as bear crawls, frog hops, or inchworms. These movements engage the core muscles while also promoting coordination and agility.

## Data Availability

Data are contained within the article.

## References

[1] Stanford M., Davie P., Mulcahy J. (2021). Growing up in the COVID-19 Pandemic: An Evidence Review of the Impact of Pandemic Life on Physical Development in the Early Years. https://www.eif.org.uk/report/growing-up-in-the-covid-19-pandemic-an-evidence-review-of-the-impact-of-pandemic-life-on-physical-development-in-the-early-years.

[2] Lazarus R. (2021). Hand-Eye Coordination. https://www.optometrists.org/general-practice-optometry/guide-to-sports-vision/vision-skills-for-sports/hand-eye-coordination/.

[3] Huggett E., Howells K. (2022). The impact of COVID-19 on the physical development of reception aged children. Phys. Educ. Matters.

[4] Department for Education (2023). Early Years Foundation Stage Statutory Framework.

[5] Veldman S.L., Chin A Paw M.J., Altenburg T.M. (2021). Physical activity and prospective associations with indicators of health and development in children aged <5 years: A systematic review. Int. J. Behav. Nutr. Phys. Act..

[6] Schmutz E.A., Leeger-Aschmann C.S., Kakebeeke T.H., Zysset A.E., Messerli-Bürgy N., Stülb K., Kriemler S. (2020). Motor competence and physical activity in early childhood: Stability and relationship. Front. Public Health.

[7] Stodden D.F., Goodway J.D., Langendorfer S.J., Roberton M.A., Rudisill M.E., Garcia C., Garcia L.E. (2008). A developmental perspective on the role of motor skill competence in physical activity: An emergent relationship. Quest.

[8] King V., Howells K. (2024). Early Years Position Paper.

[9] Jones D., Innerd A., Giles E.L., Azevedo L.B. (2021). The association between physical activity, motor skills and school readiness in 4-5-year-old children in the Northeast of England. Int. J. Environ. Res. Public Health.

[10] Office for Health Improvements and Disparities (2023). Public Health Profiles. https://fingertips.phe.org.uk/search/school%20readiness.

[11] Department for Education (2022). Help for Early Years Providers. https://help-for-early-years-providers.education.gov.uk/.

[12] Campbell S., Greenwood M., Prior S., Shearer T., Walkem K., Young S., Bywaters D., Walker K. (2020). Purposive sampling: Complex or simple? Research case examples. J. Res. Nurs..

[13] Polimac M., Vukadinovic M., Obradovic J. (2013). Differences in motor abilities of children in relation to gender and age. Exerc. Qual. Life.

[14] Navarro-Patón R., Arufe-Giráldez V., Sanmiguel-Rodríguez A., Mecías-Calvo M. (2021). Differences on motor competence in 4-year-old boys and girls regarding the quarter of birth: Is there a relative age effect?. Children.

[15] Milat A.J., King L., Bauman A.E., Redman S. (2013). The concept of scalability: Increasing the scale and potential adoption of health promotion interventions into policy and practice. Health Promot. Int..

[16] Hammer G.P., du Prel J.B., Blettner M. (2009). Avoiding bias in observational studies: Part 8 in a series of articles on evaluation of scientific publications. Dtsch. Ärzteblatt Int..

[17] U.S. Department of Health and Human Services. Centers for Disease Control and Prevention Data Collection Methods for Program Evaluation: Observation. https://www.cdc.gov/healthyyouth/evaluation/pdf/brief16.pdf.

[18] Ulrich D.A. (2000). Test of Gross Motor Development 2: Examiner’s Manual.

[19] Aye T., Oo K.S., Khin M.T., Kuramoto-Ahuja T., Maruyama H. (2017). Reliability of the test of gross motor development second edition (TGMD-2) for Kindergarten children in Myanmar. J. Phys. Ther. Sci..

[20] Cools W., De Martelaer K., Samaey C., Andries C. (2009). Movement Skill Assessment of Typically Developing Preschool Children: A Review of Seven Movement Skill Assessment Tools. J. Sports Sci. Med..

[21] Department for Education Development Mattters (2023). Non-Statutory Curriculum Gudiance for the Early Years Foundation Stage.

[22] Department for Education (2021). Statutory Framework for the Early Years Foundation Stage.

[23] Saether R., Helbostad J.L., Riphagen I.I., Vik T. (2013). Clinical tools to assess balance in children and adults with cerebral palsy: A systematic review. Dev. Med. Child Neurol..

[24] Hutchinson A.B., Yao P., Hutchinson M.R. (2016). Single-leg balance and core motor control in children: When does the risk for ACL injury occurs?. BMJ Open Sport Exerc. Med..

[25] Sallis J.F., Adlakha D., Oyeyemi A., Salvo D. (2020). An international physical activity and public health research agenda to inform coronavirus disease-2019 policies and practices. J. Sport Health Sci..

[26] van Sluijs E.M., McMinn A.M., Griffin S.J. (2007). Effectiveness of interventions to promote physical activity in children and adolescents: Systematic review of controlled trials. Br. Med. J..

[27] Jane J.Y., Burnett A.F., Sit C.H. (2018). Motor skill interventions in children with developmental coordination disorder: A systematic review and meta-analysis. Arch. Phys. Med. Rehabil..

[28] Eddy L.H., Wood M.L., Shire K.A., Bingham D.D., Bonnick E., Creaser A., Mon-Williams M., Hill L.J. (2019). A systematic review of randomized and case-controlled trials investigating the effectiveness of school-based motor skill interventions in 3- to 12-year-old children. Child Care Health Dev..

[29] Robinson L.E., Goodway J.D. (2009). Instructional climates in preschool children who are at-risk. Part I: Object-control skill development. Res. Q. Exerc. Sport.

[30] Valentini N.C., Ramalho M.H., Oliveira M.A. (2014). Movement Assessment Battery for Children-2: Translation, reliability, and validity for Brazilian children. Res. Dev. Disabil..

[31] Davies M.M. (1996). Outdoors: An important context for young children’s development. Early Child Dev. Care.

[32] van Hyfte E., Vercruysse S., Warlop G., Lenoir M. (2021). A Physical Education Program Based Upon an Obstacle Course Positively Affects Morot Competence in 6- to 7-Year-Old Children: A Pilot Study. J. Teach. Phys. Educ..

[33] Curtin D. (2023). Modeling in Physical Education. https://plt4m.com/blog/modeling-in-physical-education/#:~:text=Modeling%20is%20the%20process%20of,correctly%20and%20with%20proper%20form.

[34] Kajanus A. (2016). Physical education in Chinese schools: Role models, repetition, and winning. Educ. About Asia.

[35] Sutapa P., Pratama K.W., Rosly M.M., Ali S.K.S., Karakauki M. (2021). Improving Motor Skills in Early Childhood through Goal-Orientated Play Activity. Children.

[36] Ridgers N., Carter L.M., Stratton G., McKenzie T. (2011). Examining children’s physical activity and play behaviors during school playtime over time. Health Educ. Res..

[37] Flores F.S., Rodrigues L.P., Copetti F., Lopes F., Cordovil R. (2019). Affordances for motor skill development in home, school, and sport environments: A narrative review. Percept. Mot. Ski..

[38] Koller S.H. (2004). Ecologia do desenvolvimento humano: Pesquisa e intervenção no Brasil. Ecology of Human Development: Research and Intervention in Brazil.

[39] Gallahue D., Ozmun J.C., Goodway J.D., Goodway J. (2011). Understanding Motor Development: Infants, Children, Adolescents, Adults.

[40] NHS Southern Health, NHS Foundation Trust Handwriting and Fine Motor Skills. https://www.southernhealth.nhs.uk/our-services/a-z-list-of-services/childrens-occupational-therapy-service-isle-wight/handwriting-and-fine-motor-skills.

[41] Bremer E., Cairney J. (2018). Fundamental movement skills and health-related outcomes: A narrative review of longitudinal and intervention studies targeting typically developing children. Am. J. Lifestyle Med..

[42] Goodway D.G., Famelia R., Bakhtiar S. (2014). Future directions in physical education & sport: Developing fundamental motor competence in the early years is paramount to lifelong physical activity. Asian Soc. Sci..

[43] Howells K., Ritchie C. (2016). Chapter 10 Supporting physical development, health and well-being through the use of outdoor environments. Exploring Children’s Learning.

[44] Michigan State University (2016). Physical Development and Health. https://www.canr.msu.edu/child-family-development/school-readiness/physical-development-and-health/index.

[45] Tarmidi N.A.Z.A., Bakar K.A. (2022). The Use of Cutting Kit in Improving Young Children’s Scissor Skills. Int. J. Acad. Res. Progress. Educ. Dev..

[46] Gutek G. (2004). The Montessori Method: The Origins of an Educational Innovation: Including an Abridged and Annotated Edition of Maria Montessori’s the Montessori Method.

